# E-cigarette or Vaping Product Use Associated Lung Injury Among Three Young Adults: A Retrospective Case Series From Delaware

**DOI:** 10.7759/cureus.11031

**Published:** 2020-10-18

**Authors:** Andrew Pajak, Soraya Bascoy, Jonathan C Li, Michael Benninghoff, Andrew Deitchman

**Affiliations:** 1 Internal Medicine, ChristianaCare, Newark, USA; 2 Internal Medicine/Pediatrics Residency Program, ChristianaCare, Newark, USA; 3 Internal Medicine/Pediatrics Residency Program, University of Pittsburgh Medical Center, Pittsburgh, USA; 4 Sidney Kimmel Medical College, Thomas Jefferson University, Philadelphia, USA; 5 Department of Pulmonary & Critical Care Medicine, ChristianaCare, Newark, USA

**Keywords:** e-cigarette, evali, vaping, c-reactive protein, corticosteroids, internal medicine-pediatrics, lung disease, biomarker

## Abstract

Background: E-cigarette or vaping associated lung injury (EVALI) is a lung disease associated with an inflammatory response to the vaping fluid. Currently, diagnosis remains elusive without definitive biomarkers.

Case presentation: Herein, we describe three cases of EVALI among 18- to 21-year-old patients ranging from mild to severe. All cases presented with a combination of respiratory, gastrointestinal, and constitutional symptoms. Oxygen support and level of medical care varied based on disease severity. Bilateral pulmonary opacities were observed on chest imaging in each case. Additionally, each case had markedly elevated inflammatory markers, specifically C-reactive protein (CRP). None of these patients improved with intravenous (IV) antibiotics and all required IV corticosteroid therapy to achieve clinical improvement.

Conclusion: EVALI should be suspected among young, otherwise healthy patients who present with new-onset hypoxia, non-specific gastrointestinal symptoms, and endorse a history of vaping. Though considered a diagnosis of exclusion, diagnosing EVALI requires thorough history taking. Inflammatory studies, CRP, and erythrocyte sedimentation rate (ESR) should be considered adjunctive biomarkers to aid clinicians when the diagnosis remains unclear. Corticosteroids are the mainstay of treatment and patients should have close follow-up whether or not they require hospitalization.

## Introduction

E-cigarette or vaping associated lung injury (EVALI) is a lung disease associated with an inflammatory response to the components of the vaping fluid. The use of vape “pens” has been increasing in popularity over the past decade particularly among adolescents and young adults [1]. The first reports identifying EVALI occurred in July 2019. By the end of August, approximately 120 cases had been confirmed across 16 states and continued to rise to over 500 cases across 39 states by mid-September [2,3]. As of January 21, 2020, the Centers for Disease Control and Prevention (CDC) has reported over 2,700 hospitalizations and 60 deaths [4].

Scientific evidence regarding the effects of vaping on human health is limited. Despite vaping delivering fewer combustion byproducts and toxins than cigarettes, comparison studies on harm reduction remain inconclusive [5]. While vape pens were initially designed and marketed as smoking cessation aids/cigarette alternatives, these products continued to lack standardization and regulation by the FDA, both of which are factors that render these products popular among youth [5]. Nevertheless, multiple compounds have been identified in the vapor including flavorants, volatile organic compounds, and heavy metals, all of which are known respiratory irritants and toxins [6,7]. The most consistent compound identified in association with EVALI, however, is vitamin E acetate, which is primarily used in tetrahydrocannabinol (THC)-containing cartridges [8].

A unifying pathologic process has yet to be identified. Studies thus far have reported a variety of histopathologic and radiologic findings which, in summation, support a non-fibrotic, inflammatory pattern of lung injury [9-11]. Diagnostic use of inflammatory markers in the context of EVALI is not well described in the literature. We present three cases of EVALI among 18- to 21-year-old patients ranging from mild to severe in presentation, all with markedly elevated inflammatory markers, and briefly discuss the diagnostic utility of these markers.

## Case presentation

Case 1

**Figure 1:**
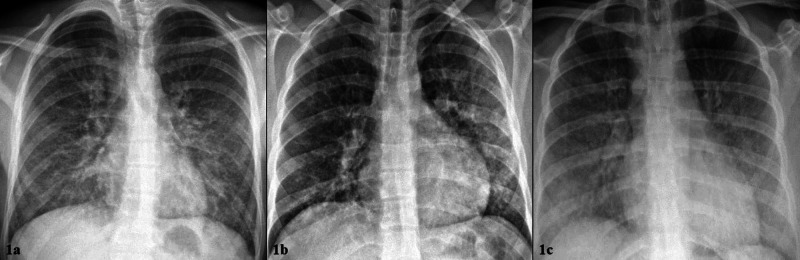
a-c: Initial chest X-ray at presentation. From left to right: Patient 1, Patient 2, Patient 3. 1a “Extensive bilateral ground glass and interstitial lung opacities.” 1b “Extensive bilateral patchy opacities, greater in the left lung.” 1c “Diffuse, bilateral, ground glass lung opacities with hazy appearance, vascular congestion.”

**Figure 2:**
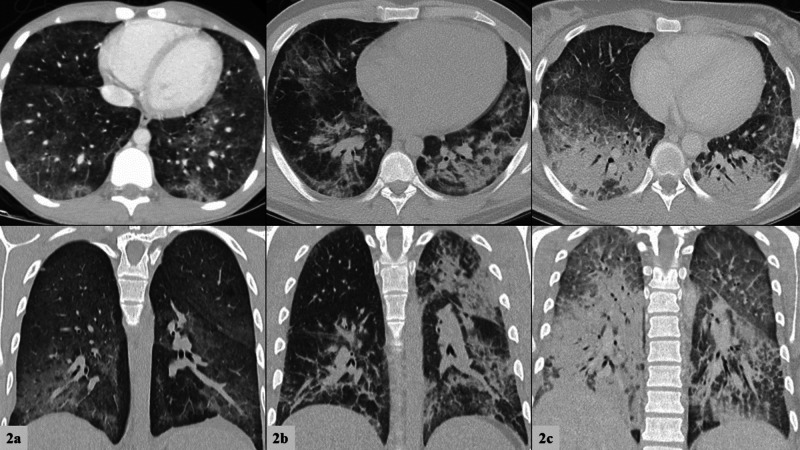
a-c: Initial computed tomography of the chest. Top row frames depict transverse views and bottom row frames depict coronal views. From left to right: Patient 1, Patient 2, Patient 3. 2a “Ill-defined fluffy confluent centrilobular nodules seen bilaterally, more confluent and dense the lung bases with subpleural sparing.” 2b “Bilateral patchy ground glass and consolidative opacities.” 2c “Dense bilateral lower lobe consolidations. Bilateral upper lobe and right middle lobe ground glass consolidations with interstitial thickening.”

An 18-year-old male with no significant past medical history presented to the emergency department (ED) after two weeks of worsening dyspnea with new abdominal pain, severe nausea, vomiting, and cough. On presentation he was febrile (39.2^o^C), tachycardic (127 beats/minute), and hypoxemic to 86% on ambient air. Supplemental oxygen with 2L nasal cannula (NC) was required. Chest x-ray (CXR) revealed extensive bilateral opacities (Figure 1a). Bloodwork was significant for leukocytosis to 18,800/nl (ref: 3.9-10.6/nl) with 93.7% (ref: 50-60%) neutrophilic predominance, an elevated total bilirubin to 1.4mg/dL (ref: 0.2-1.0) without transaminitis, and a procalcitonin of 4.73ng/mL (ref: 0.0-0.24ng/mL). Computed tomography (CT) of the chest with contrast showed, “ill-defined fluffy confluent centrilobular nodules seen bilaterally, more confluent and denser at the lung bases with sub-pleural sparing” (Figure 2a). He was empirically treated for community-acquired pneumonia with azithromycin and ceftriaxone for three days but failed to improve clinically with persisting fevers and hypoxemia. Blood cultures and studies for common viral respiratory pathogens were negative. On day three, lab work revealed significant elevations in CRP to 27.9mg/dL (ref: 0-.8 mg/dL) and ESR to 102mm/hr (ref: 0-15mm/hr). Additional history revealed the patient vapes at least three times weekly using THC-containing products from an unlicensed dealer. EVALI was suspected and the patient was initiated on methylprednisolone 50mg daily. Over the next three days, he showed clinical improvement and was subsequently afebrile, and no longer required supplemental oxygen. Hospital day five laboratory data demonstrated the resolution of his leukocytosis and improvement in CRP to 16.2mg/dL. The patient was discharged home on hospital day six on a prednisone taper regimen. 

Case 2

A previously healthy 20-year-old male presented to an urgent care facility after one week of cough, nausea, vomiting, and diarrhea. He was diagnosed with viral gastroenteritis and sent home with ondansetron. Three days later, he presented to our emergency department for persistent symptoms. Vital signs were significant for tachycardia (106 beats/minute) and oxygen saturation of 91% on ambient air. He displayed conversational dyspnea which improved on 2L NC. CXR revealed extensive bilateral patchy opacities, worse in the left lung base (Figure 1b). Laboratory data were significant for leukocytosis to 21,200/nl with 90.0% neutrophilic predominance, an elevated total bilirubin to 1.9mg/dL without transaminitis, and procalcitonin to 0.54ng/mL (ref: 0.0-0.2 ng/mL). CT of the chest with contrast revealed, “bilateral patchy ground-glass and consolidative opacities” (Figure 2b). A viral respiratory pathogen panel was positive for rhinovirus. Empiric treatment for bacterial pneumonia was initiated with ceftriaxone and doxycycline. On day three of hospitalization, he had clinical deterioration and required 6L NC to maintain oxygen saturation above 90%. CRP was obtained and found to be significantly elevated to 42.9mg/dL. Additional history revealed a habit of daily vaping with both commercially obtained nicotine products and THC-containing products from an unlicensed dealer. He was given intravenous methylprednisolone 125mg once, followed by 60mg twice a day dosing thereafter for the treatment of suspected EVALI. CRP was not repeated, however, procalcitonin was repeated and decreased to 0.15ng/mL after steroid therapy. Four days after initiation of steroid therapy his hypoxemia resolved and no longer required supplemental oxygen. He was discharged on hospital day six with a prednisone taper.

Case 3

**Figure 3:**
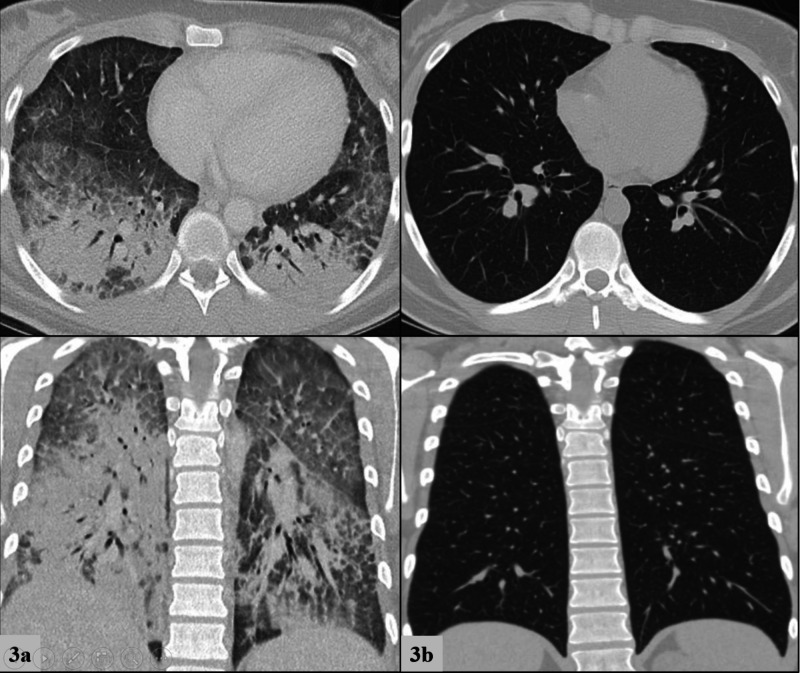
Chest computed tomography for Patient 3 at presentation (3a, left column) compared to 1 month after discharge (3b right column). Top row frames depict transverse views and bottom row frames depict coronal views.

A 20-year-old healthy female presented to our emergency department with seven days of worsening shortness of breath, cough, vomiting, decreased oral intake, and three days of documented fever. The patient had been previously seen in two different acute care facilities the week prior. In the first facility, she was diagnosed with anxiety and prescribed benzodiazepines; in the second, she was prescribed a methylprednisolone dose pack for a viral respiratory tract infection. Both treatments were discontinued shortly into their course, as her symptoms worsened, prompting her to present in our ED. Review of systems was negative for any recent viral illness, travel, or sick contacts, but did uncover a history of vaping THC-containing e-cigarettes daily for at least one and a half years. On presentation she was febrile (38.9^o^C), tachycardic (147 beats/minute), normotensive, tachypneic (27 breaths/minute), and requiring 15L by non-rebreather mask to maintain oxygen saturation above 90%. She was in moderate respiratory distress. Physical examination was additionally notable for scleral icterus. Her laboratory data demonstrated a leukocytosis to 13,000/nl with neutrophilic predominance, mild transaminitis, total bilirubin of 2.6mg/dL, alkaline phosphatase (ALP) of 149mg/dL, prothrombin time (PT) of 18.1 seconds, and international normalized ratio (INR) of 1.6. CXR demonstrated diffuse, bilateral infiltrates (Figure 1c) and follow-up CT chest with contrast showed bilateral lower lobe consolidations, along with bilateral upper and middle lobe ground-glass opacities (Figure 2c). She was admitted to the intensive care unit and required supportive care with high flow nasal cannula delivering up to 50L of flow and 100% inspired fraction of oxygen. There was high clinical suspicion for EVALI, so the patient was immediately started on methylprednisolone 60mg every six hours daily and empiric antibiotics for possible community-acquired pneumonia. Subsequent lab work on hospital day three showed significantly elevated CRP to 23.8mg/dL. Her clinical status continued to improve and a repeat CRP on hospital day five had decreased to 6.0mg/dL. She was weaned off supplemental oxygen support in 96 hours and was discharged home on a prednisone taper. Repeat CT of the chest one month after discharge revealed resolution of pulmonary infiltrates and consolidations (Figure 3). The key components of each case are compared below in Table 1. 

**Table 1:** Patient case summaries presented for comparison. NC: nasal cannula, HFNC: high-flow nasal cannula, TCH: tetrahydrocannabinol

	Patient 1 (18 YO M)	Patient 2 (20 YO M)	Patient 3 (20 YO F)
Vape history	3x/week Cartridges from unlicensed dealer	Daily Cartridges commercially purchased and THC-cartridges from unlicensed dealer	Daily for 1.5 years
Treated outpatient for viral process	No	Yes	Yes
Symptoms: Resp: GI: Systemic:	Cough Diffuse abdominal pain, severe nausea, vomiting, anorexia None	Cough Nausea, vomiting, diarrhea None	Cough, dyspnea on exertion Mild epigastric discomfort Intermittent fevers
Maximum Oxygenation	Hypoxic; 92% on 2L NC	Hypoxic; 91% on 6L NC	Hypoxic; 94% on 50L/min 100% HFNC
Urine toxicology	Cannabis	Cannabis	Cannabis, opioid
Infectious workup	Noncontributory	+Rhinovirus	Noncontributory
Steroid initiation	Day 3	Day 3	Day 1
Hospital duration	6 days	6 days	6 days
Follow-up	None	None	1 month

## Discussion

These cases illustrate the salient features of EVALI reported in the literature and present unique findings for discussion. Large surveillance studies from the CDC show that most patients are: male, less than 35 years old, and report the use of THC-containing products [8,12,13]. Most patients present with pulmonary, gastrointestinal, and constitutional symptoms [2]. Infectious etiologies should be considered first as EVALI remains a diagnosis of exclusion. When suspected, a detailed vaping/e-cigarette use history should be obtained and include substances vaped, vendor, brand, duration and frequency of use, and time of last use. The recommended initial workup includes complete blood count (CBC), complete metabolic panel (CMP), urine toxicology screen, and chest x-ray, with consideration for CT scan of the chest [14,15].

Uniquely, in this series, we observed markedly elevated inflammatory markers, CRP and ESR, as well as procalcitonin and bilirubin. The presence and/or degree of elevation may have utility in differentiating inflammatory lung pathologies, specifically vaping associated lung injury. CRP was elevated in each case, ranging from 23.8-42.9mg/dL. Their levels improved only after initiating steroid therapy and correlated with clinical improvement and disease resolution. There is limited data regarding the use of CRP in diagnosing EVALI. In one EVALI case series of six patients, Maddock et al. reported elevated CRP values ranging from 20.4-30.7mg/dL, and also elevated ESR from 60-128mm/hr [11]. In another case series with six patients, Triantafyllou et al. also reported elevated CRP values in each of their patients, with a mean value of 28.5mg/dL and a mean ESR of 84 mm/hr [16]. To our knowledge, there have not been any reports on CRP values in relation to treatment modalities and clinical course. 

**Figure 4:**
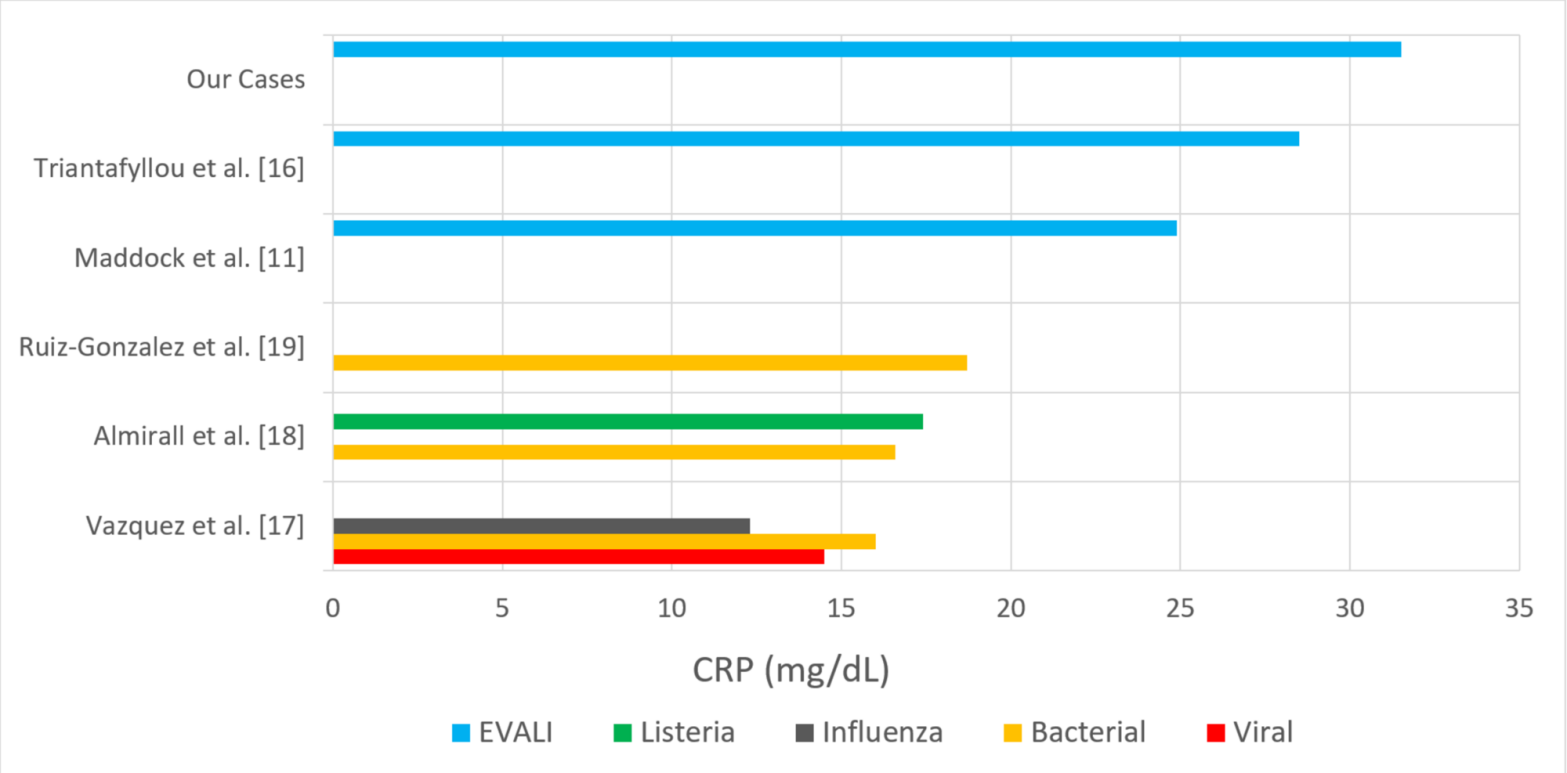
Comparison of reported mean C-reactive protein (CRP) values (mg/dL) of non-influenza viral, influenza, bacterial, and Listeria pneumonias to e-cigarette or vaping associated lung injury (EVALI).

When CRP values measured in EVALI are compared to CRP values measured in cases of bacterial, viral, or atypical pneumonias, there is a notable difference in elevation that has not yet been reported in the literature. For comparison, Vazquez et al. reported a mean CRP of pyogenic pneumonia at 16mg/dL, non-influenza viral pneumonia at 14.45mg/dL, atypical pneumonia at 12.64mg/dL, and influenza at 12.3mg/dL [17]. Additionally, Almirall et al. reported mean CRP values of pneumococcal pneumonia at 16.6mg/L and *Listeria* pneumonia at 17.8 mg/dL [18]. Lastly, Ruiz-Gonzalez et al. conducted a prospective analysis of 923 patients admitted to a large teaching hospital in Lleida, Spain in which they reported a mean CRP of 18.7mg/dL (range 12.3-27.8 mg/dL) for bacterial pneumonia infections [19]. When comparing those reviewed studies to our findings and those by Maddock et al. and Triantafyllou et al., the CRP value in vaping associated lung injury is consistently more elevated than that of pyogenic and viral pneumonias (Figure 4) [11,16]. One hypothesis for the larger elevation of CRP in EVALI is it may be a marker for a more diffuse lung injury and inflammatory pattern when compared to focal pneumonias and furthermore may serve as a potential differentiating biomarker in EVALI. 

Procalcitonin was obtained and elevated in two of three. We did not observe a positive relationship between the degree of procalcitonin elevation and severity of disease. This observation held true for CRP as well. Therefore, these biomarkers may be useful in supporting diagnosis and monitoring for a response to treatment. Finally, since EVALI tends to present similar to viral illness (non-specific systemic symptoms), procalcitonin may be useful in ruling out a viral pneumonia, but will not be useful in differentiating EVALI from a bacterial process. Lastly, we observed elevated bilirubin in each case, despite transaminase values within normal limits. As mentioned previously, there have been reports of up to 50% of patients having elevated liver function tests (LFTs), but no reports of hyperbilirubinemia and its utility in EVALI diagnosis. We suspect this is a reflection of systemic inflammation.

**Figure 5:**
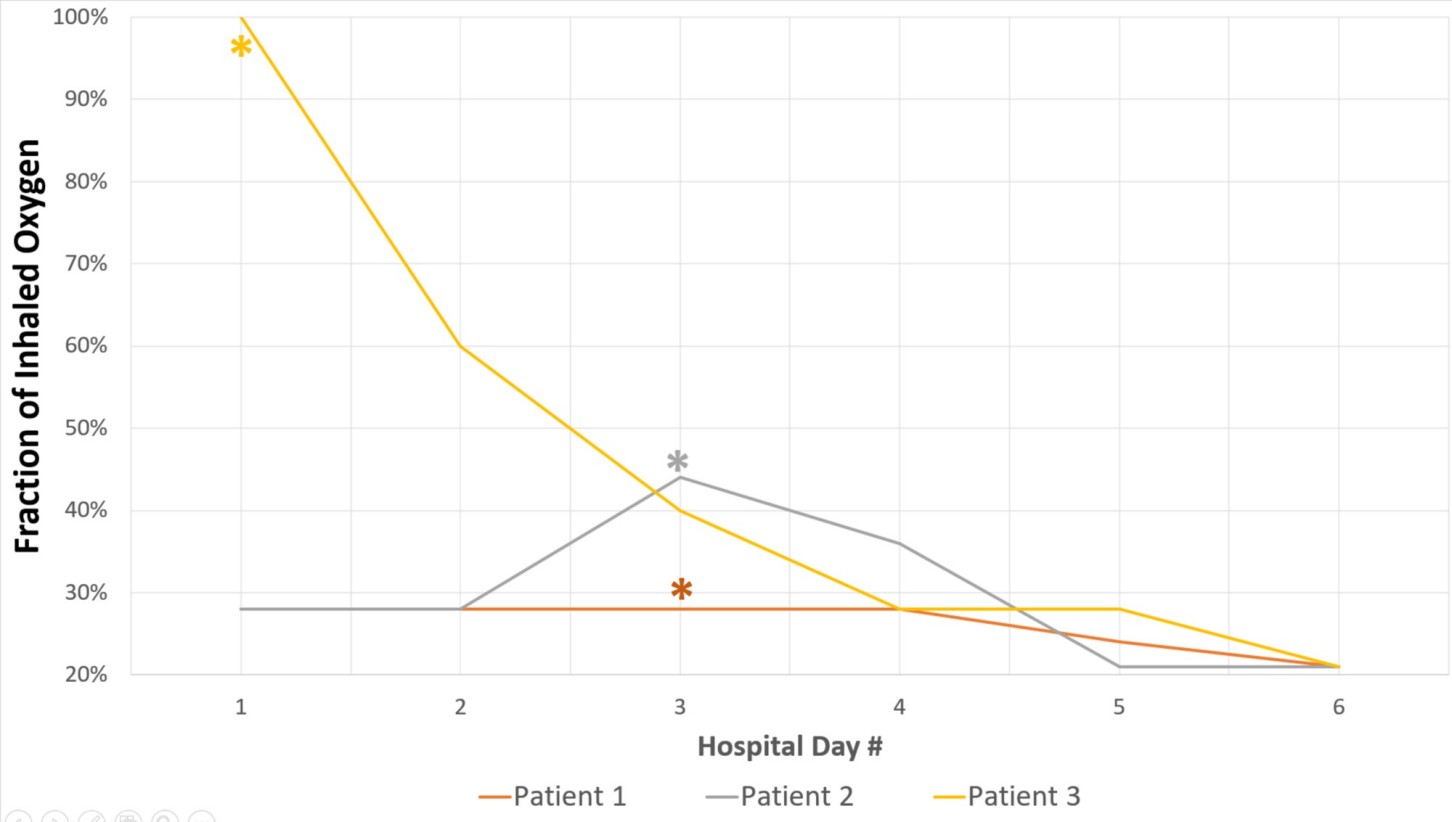
Reduction in oxygen support requirements in relation to steroid therapy during hospital course. Asterisk (*) denotes initiation of corticosteroid therapy.

**Figure 6:**
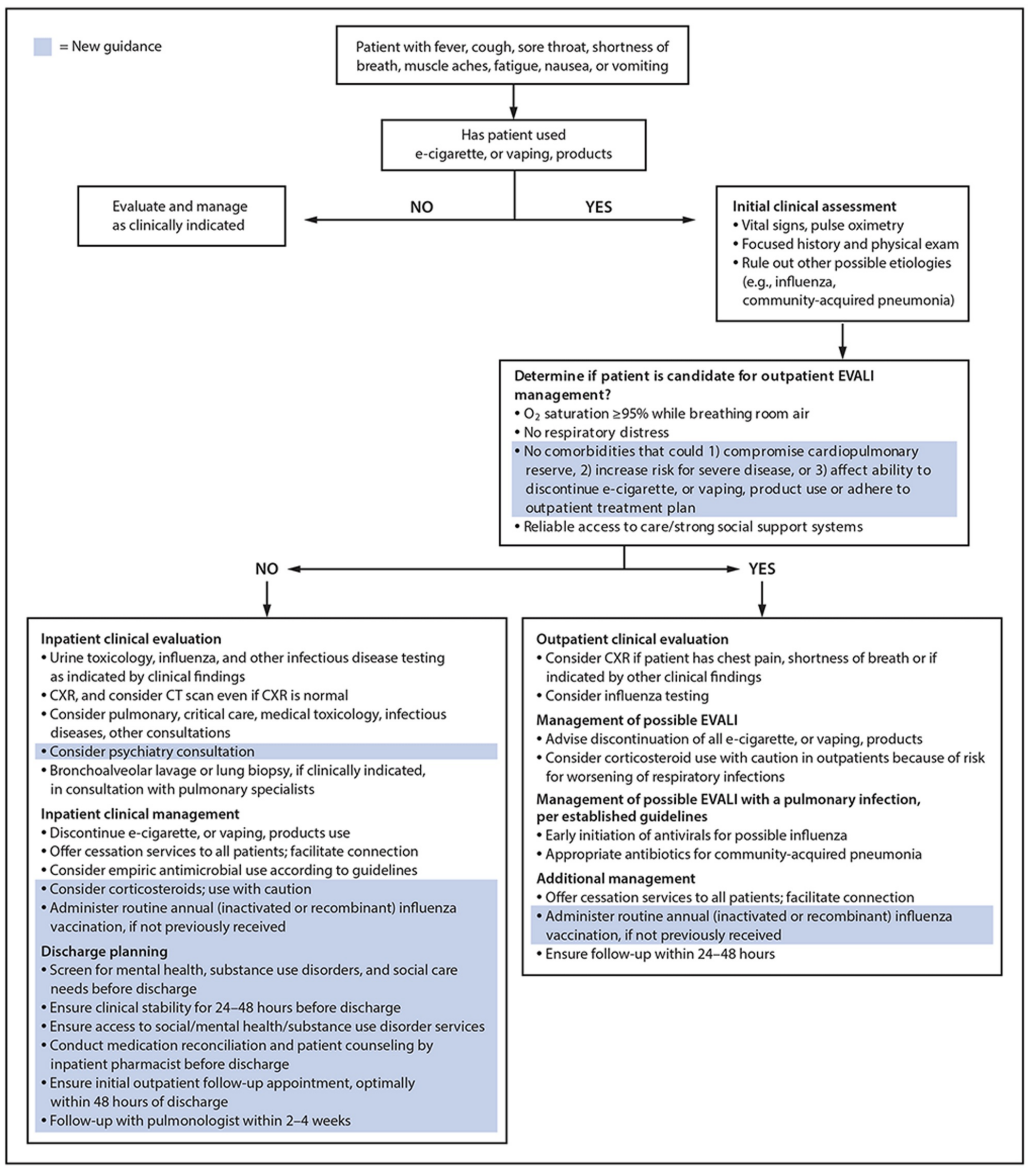
The Centers for Disease Control and Prevention (CDC) recommended approach to workup and management of e-cigarette or vaping associated lung injury (EVALI).

Treatment guidelines and initial dosing regimens for EVALI remain debated but there is consensus among practitioners regarding general management strategies. Recommendations include treatment for pneumonia and influenza when appropriate, consideration of corticosteroids with dosing ranging from 0.5-1mg/kg when patients fail to improve on antimicrobial treatment, 24- to 48-hour follow-up for patients not admitted to the hospital, and discontinuation of vaping/e-cigarette use. Among our patients, each was treated with empiric antibiotics but did not begin to show improvement in oxygen requirements until the initiation of corticosteroids (Figure 5). Corticosteroids should be initiated both inpatient and continued as a prednisone taper upon discharge. Discharge is recommended after 48 hours of stable vital signs on ambient air as 25% of readmissions and deaths occur within 48 hours of discharge; therefore, close follow-up is warranted. Additionally, follow-up with a pulmonologist should occur two to four weeks after discharge to assess lung function and for radiographic resolution of disease [14,15]. All cases should be reported to the state health department. A workup schema developed by the CDC is presented in Figure 6 [14,15].

Despite many recent advances in our understanding of EVALI, there is still much to be learned about this entity in medicine. E-cigarette use is on the rise, particularly among adolescents and young adults, with approximately 35% reporting prior use in 2019 according to the National Youth Tobacco Survey [1,20]. There remain many chemicals involved in the production of vaping liquids to deliver both nicotine and THC [4,20]. Although vitamin E acetate has emerged as the main driver behind recent cases of EVALI, there is still a lack of evidence showing other vapor components and devices to be safe [4,8,20]. Until the health effects of e-cigarettes and e-cigarette components can be thoroughly investigated, some countries such as Canada have enacted its federal government to oversee its use and distribution. At this time more research needs to be conducted weighing the short- and long-term outcomes of e-cigarette use as well as establishing health policy to best protect our population [4].

## Conclusions

EVALI remains a diagnosis of exclusion and a challenging condition to identify. Presentation includes both respiratory and gastrointestinal symptoms, with the possibility of acute respiratory decompensation requiring intensive care treatment. Workup typically reveals a neutrophilic leukocytosis and a nonspecific diffuse process on pulmonary imaging. Inflammatory markers, specifically ESR and CRP, may aid in the diagnosis of EVALI and differentiation from various pneumonias. Trending these biomarkers throughout hospitalization may be useful in assessing response to therapy and should be considered in all suspected cases of EVALI. Further investigations of CRP and ESR are needed to better understand their role in aiding clinicians in making the correct diagnosis. Currently, corticosteroids remain the mainstay of treatment, with methylprednisolone dosed from 0.5-1mg/kg daily with a gradual taper over five to 10 days. Close follow-up after discharge is warranted due to a high risk of hospital readmission or death. As e-cigarette use continues to rise, improved understanding of short- and long-term impacts is needed as well as policies to best protect the health of users.
